# Recombinant Tissue Plasminogen Activator (r-tPA) Induces In-Vitro Human Neutrophil Migration via Low Density Lipoprotein Receptor-Related Protein 1 (LRP-1)

**DOI:** 10.3390/ijms21197014

**Published:** 2020-09-23

**Authors:** Luca Liberale, Maria Bertolotto, Silvia Minetti, Paola Contini, Daniela Verzola, Pietro Ameri, Giorgio Ghigliotti, Aldo Pende, Giovanni G. Camici, Federico Carbone, Fabrizio Montecucco

**Affiliations:** 1 Center for Molecular Cardiology, University of Zürich, Wagistrasse 12, 8952 Schlieren, Switzerland; luca.liberale@uzh.ch (L.L.); giovanni.camici@uzh.ch (G.G.C.); 2 First Clinic of Internal Medicine, Department of Internal Medicine, University of Genoa, 6 Viale Benedetto XV, 16132 Genoa, Italy; maria.bertolotto@unige.it (M.B.); silvia.minetti@unige.it (S.M.); federico.carbone@unige.it (F.C.); 3 Clinical Immunology, Department of Internal Medicine, University of Genoa, 6 viale Benedetto XV, 16132 Genoa, Italy; paola.contini@unige.it; 4 Division of Nephrology, Dialysis and Transplantation, Department of Internal Medicine, University of Genoa, 6 Viale Benedetto XV, 16132 Genoa, Italy; daniela.verzola@unige.it; 5 IRCCS Ospedale Policlinico San Martino Genoa—Italian Cardiovascular Network, 10 Largo Benzi, 16132 Genoa, Italy; pietroameri@unige.it (P.A.); gghiglio@unige.it (G.G.); apende@unige.it (A.P.); 6 Laboratory of Cardiovascular Biology, IRCCS Ospedale Policlinico San Martino & Department of Internal Medicine, University of Genoa, 16126 Genoa, Italy; 7 Clinic of Emergency Medicine, Department of Emergency Medicine, University of Genoa, 16126 Genoa, Italy

**Keywords:** tissue plasminogen activator, neutrophil, inflammation

## Abstract

Thrombolysis is the gold standard treatment for acute ischemic stroke. Besides its fibrinolytic role, recombinant tissue plasminogen activator (r-tPA) holds several non-fibrinolytic functions. Here, we investigated the potential role of r-tPA on human primary neutrophil migration in vitro. By means of modified Boyden chamber migration assay and checkerboard analysis we showed a dose-dependent chemotactic effect of r-TPA with a maximum effect reached by 0.03 mg/mL (0.003–1 mg/mL). Pre-incubation with MAP kinases inhibitors allowed the identification of PI3K/Akt, but not ERK1/2 as the intracellular pathway mediating the observed effects. Furthermore, by means of real-time PCR, immunocytochemistry and cytofluorimetry we demonstrated that the r-tPA receptor low density lipoprotein receptor-related protein 1 (LRP-1) is synthetized and expressed by neutrophils in response to r-tPA and TNF-α. Inhibition of LRP-1 by receptor-associated protein (RAP), prevented r-tPA-mediated F-actin polymerization, migration and signal through Akt but not ERK1/2. Lastly, also neutrophil degranulation in response to r-tPA seems to be mediated by LRP-1 under adhesion conditions. In conclusion, we show that r-tPA induces neutrophil chemotaxis through LRP-1/Akt pathway. Blunting r-tPA-mediated neutrophil activation might be beneficial as an adjuvant therapy to thrombolysis in this setting.

## 1. Introduction

Cerebro- and cardiovascular acute ischemic afflictions such as myocardial infarction and ischemic stroke benefit of early reperfusion to reduce organ damage. Together with interventional approaches, reperfusion strategies include the infusion of fibrinolytic agents such as recombinant tissue plasminogen activator (r-tPA). Currently, r-tPA represents the only medical therapy to improve outcomes in patients with acute ischemic stroke and its intravenous injection is recommended under specific conditions by international guidelines [1,2]. Yet, the late restoration of the blood flow in a previously ischemic tissue associates with a paradoxical exacerbation of inflammation and oxidative stress leading to cellular dysfunction and death, the so-called ischemia/reperfusion (I/R) injury [3]. 

Beside their important roles in host defence against infectious agents, neutrophils have been implicated in the pathogenesis of different conditions characterized by sterile inflammation such as I/R injury [4,5]. After blood flow re-establishment, circulating neutrophils are recruited early within the tissue and concur to its damage by different mechanisms including release of pro-inflammatory and pro-oxidant mediators such as interleukins and reactive oxygen species, degranulation of different proteases including matrix metalloproteases (MMPs), myeloperoxidase (MPO) and neutrophil elastase or formation of neutrophil extracellular traps [6]. 

Of interest, together with its direct action on vascular patency, r-tPA was also shown to contribute to I/R injury indirectly by inducing neutrophil degranulation both in-vitro and in-vivo [7]. Yet, the effect of r-tPA on neutrophil function remains only partially investigated, specifically the receptors and pathways involved in such process have not been fully identified. This information will be of utmost importance to counteract r-tPA-induced neutrophil activation potentially widening our interventional options in the setting of I/R injury and ischemic stroke. Accordingly, in this study, we investigated the role of r-tPA in human primary neutrophil migration. Furthermore, we characterized the membrane receptor and the intracellular pathways underlying the observed effect. 

## 2. Results

### 2.1. r-tPA Induces Dose-Dependent Migration of Human Neutrophils 

**Figure 1:**
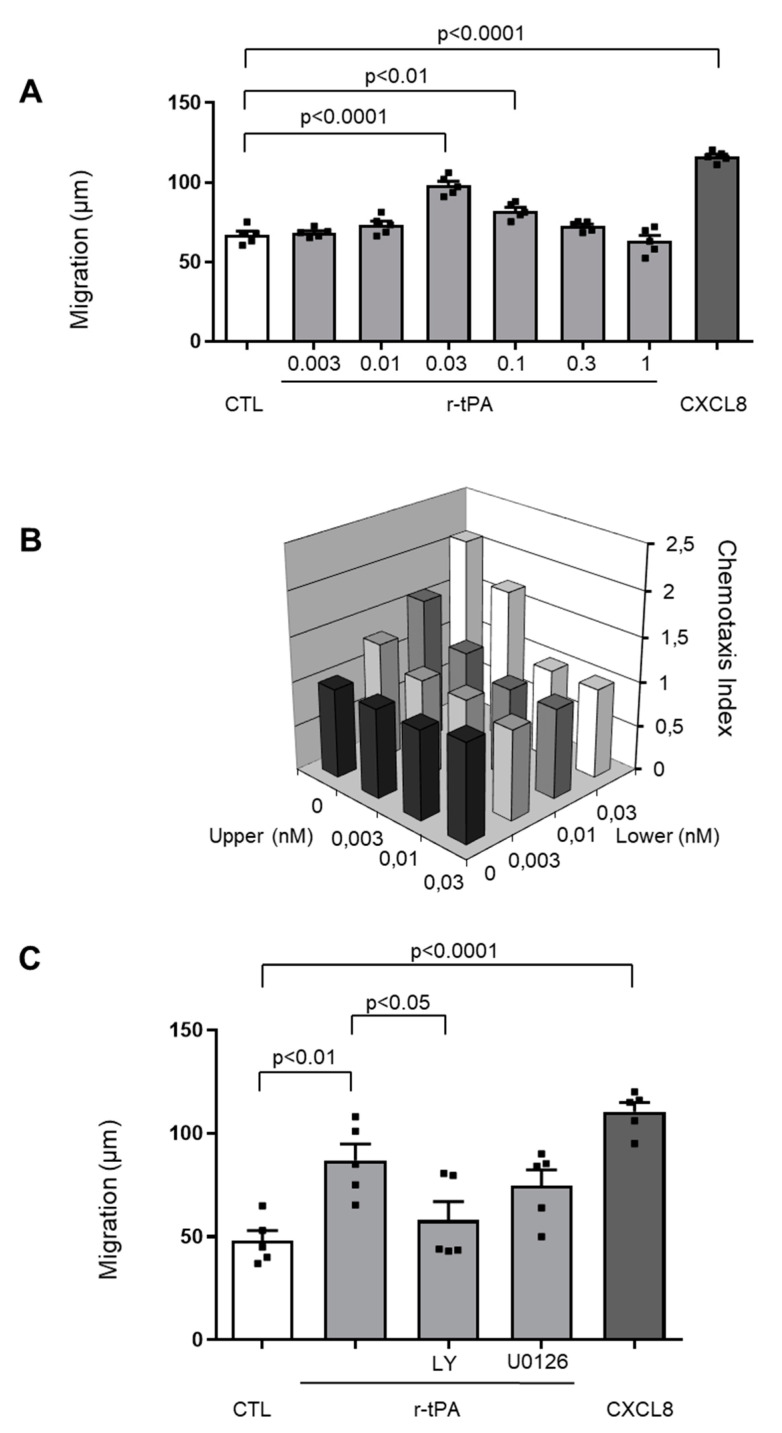
Recombinant tissue plasminogen activator (r-tPA) induces dose-dependent migration in human neutrophils. (**A**) The graphic represents the migration assay of neutrophils towards control medium (CTL), r-tPA (0.003 mg/mL up to 1 mg/mL) or 1 nM CXCL8 as positive control. Data are expressed as mean of migration distance (μm) ±1 SEM in the nitrocellulose assay, *n* = 5. (**B**) Checkerboard analysis showing that neutrophil migration depends on a r-tPA gradient across the filter. Untreated monocytes were seeded with increasing concentrations of r-tPA in the upper well, increasing concentrations of r-tPA were also added to the lower well. Data are expressed as mean of chemotaxis index in the polycarbonate assay, *n* = 5. (**C**) Pre-treatment with PI3K Inhibitor reduces neutrophils migration towards r-tPA. Human neutrophils were pre-treated for 1 h with control medium (CTL) or 10 µM LY294002 (PI3K inhibitor, LY) or 10 µM U0126 (MEK 1/2 inhibitor). Then their chemotactic function was tested towards 0.03 mg/mL r-tPA or 1nM CXCL8 as positive control. Data are expressed as mean of migration distance (μm) ±1 SEM in the nitrocellulose assay, *n* = 6.

Locomotory function of freshly isolated human neutrophils from healthy volunteers was tested in response to r-tPA employing the modified Boyden chamber assay with nitrocellulose filter. Concentrations of r-tPA herein employed are within the range observed in serum of patients undergoing thrombolysis [8]. Neutrophil migration toward different doses of r-tPA displayed the typical bell-shaped dose-response graphic of chemoattractants (Figure 1A). The magnitude of neutrophil migration induced by 0.03 mg/mL r-tPA was similar to that generated by 1 nM chemokine (C-X-C motif) ligand 8 (CXCL8) (Figure 1A), suggesting for r-tPA a powerful locomotion-inducing activity. To understand whether the migration was due to the induction of chemotaxis or to chemokinesis, we performed a checkerboard analysis with increasing concentrations of r-tPA above and below the filter (Figure 1B). A directed migration vs. a gradient across the filter rather than random migration was observed indicating that r-tPA induces chemotaxis rather than random chemokinesis. Lastly, to investigate the signaling pathways involved in neutrophils migration towards r-tPA, we performed experiments with selective kinase inhibitors for PI3K and MEK1/2 (ERK1/2 activator). Figure 1C shows that pre-incubation with 10 µM LY294002 (PI3K inhibitor) significantly reduced r-tPA-induced neutrophil migration. On the opposite, neutrophil pre-treatment with the MEK1/2 inhibitor U0126 had no effect on their migration (Figure 1C).

### 2.2. Low-Density Lipoprotein Receptor-Related Protein-1 (LRP-1) Expression on Human Neutrophils

**Figure 2:**
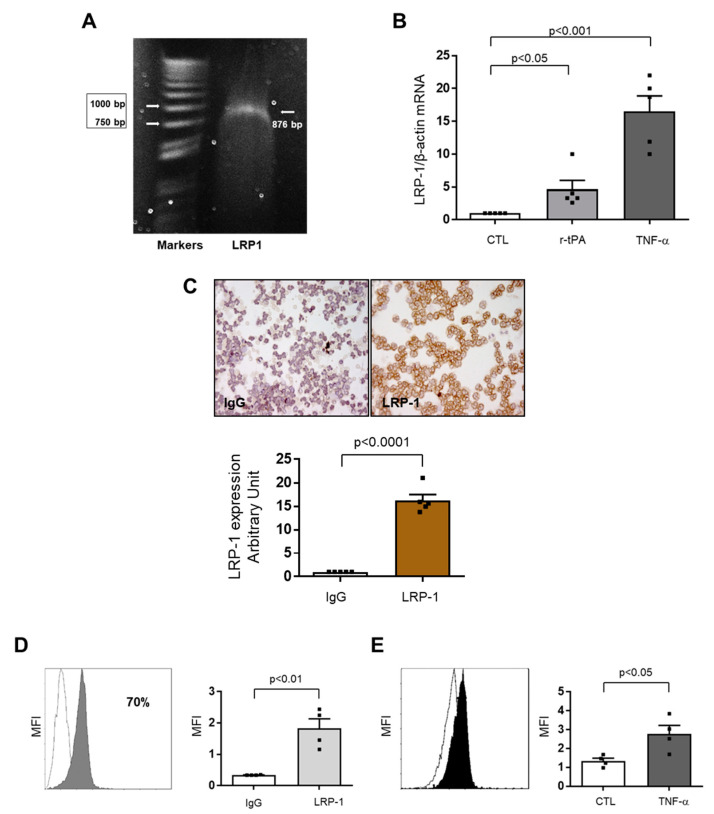
Low density lipoprotein receptor-related protein 1 (LRP1) is expressed on human neutrophil membrane. (**A**) Representative agarose gel of PCR products from human neutrophils showing an 876 amplicon. On the left, the molecular weights of the markers are shown. (**B**) Recombinant tissue plasminogen activator (r-tPA) and tumor necrosis factor (TNF)-α increase LRP-1 mRNA expression as assessed by real-time RT-PCR: cells were stimulated in presence or absence of 0.1 mg/mL r-tPA or 200 U/mL of TNF-α. All data were first normalized to β-actin mRNA and values were expressed as fold increase ±1 SEM vs. untreated (CTL) cells, *n* = 5. (**C**) Immunocytochemistry of human neutrophil. Representative images of LRP-1 staining and densitometric analysis of digital images. Data are expressed as mean ±1 SD, *n* = 5. (**D**) Flow cytometry analysis of human neutrophils. Grey profile: LRP-1 expression of human neutrophils freshly purified from peripheral blood and labelled with anti-LRP-1; white profile: neutrophils freshly purified from peripheral blood and labelled with an isotype-matched negative control antibody. Results were expressed as median fluorescence intensity, *n* = 4. (**E**) TNF-α increases LRP-1 expression. Flow cytometry analysis: human neutrophils stimulated with medium alone (CTL, white profile) or 200 U/mL of TNF-α (black profile) and labelled with the anti-LRP-1 antibody. Results were expressed as median fluorescence intensity (MFI: ±1 SEM), *n* = 4.

The scavenger receptor low density lipoprotein receptor-related protein 1 (LRP-1) is a transmembrane receptor that was previously reported to work as a membrane receptor for tPA [9]. *LRP1* mRNA was detectable in freshly isolated neutrophils by mean of PCR (Figure 2A), furthermore treatment with 0.1 mg/mL of r-tPA and 200 U/mL of tumour necrosis factor (TNF)-α induce *LRP1* transcription by five and 17 times, respectively (Figure 2B). The expression of LRP-1 at the protein level was then confirmed with different techniques. First, the presence of LRP-1 on the membrane of unstimulated neutrophils was confirmed by immunocytochemistry staining vs. a negative isotype control (Figure 2C). Next, flow cytometry analysis showed the evident expression of this receptor on the membrane of neutrophils (70% positive cells as compared with isotype-matched antibody) (Figure 2D). In line with the gene expression levels, flow cytometry analysis confirmed an increase of mean fluorescence intensity (MFI) by 2-times in neutrophils stained with anti-LRP-1 antibody and pre-treated with TNF-α (Figure 2E). 

### 2.3. Neutrophil Migration towards r-tPA Is LRP-1-Dependent

**Figure 3:**
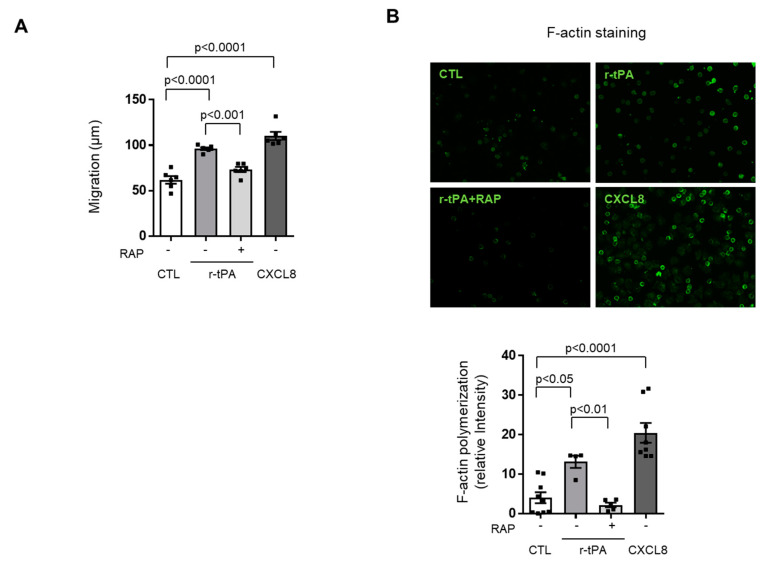
Pre-treatment with receptor-associated protein (RAP) inhibits neutrophils migration and F-actin polymerization towards recombinant tissue plasminogen activator (r-tPA). (**A**) Human neutrophils were pre-treated for 1 h with control medium (CTL) or 0.5 µM RAP and tested for their chemotactic response to 0.03 mg/mL r-tPA and 1nM CXCL8 as positive control. Data are expressed as mean of migration distance (μm) ±1 SEM in the nitrocellulose assay, *n* = 6. (**B**) F-actin content in neutrophils: tests were performed under the same experimental conditions as abovementioned. Representative pictures showing F-actin stained in green and quantification of the images. Data are expressed as relative intensities (mean ± 1 SEM), *n* = 4–9.

Receptor-associated protein (RAP) universally inhibits ligand interaction with low-density lipoprotein (LDL) receptor family including LRP-1 [9]. In order to investigate whether r-tPA-induced chemotaxis was depending on LRP-1, neutrophils were pre-incubated with 0.5 µM RAP or control medium before Boyden chamber experiments. As a result, RAP could significantly inhibit neutrophil migration toward r-tPA (Figure 3A). In accordance with the above, the significant induction of F-actin polymerization due to the presence of r-tPA as compared to control medium was completely reversed by neutrophil pre-incubation with the LRP-1 inhibitor RAP; CXCL8 served as the positive chemoattractant control (Figure 3B).

### 2.4. LRP-1 Regulates Neutrophil Migration towards r-tPA via Akt, but Not ERK ½

**Figure 4:**
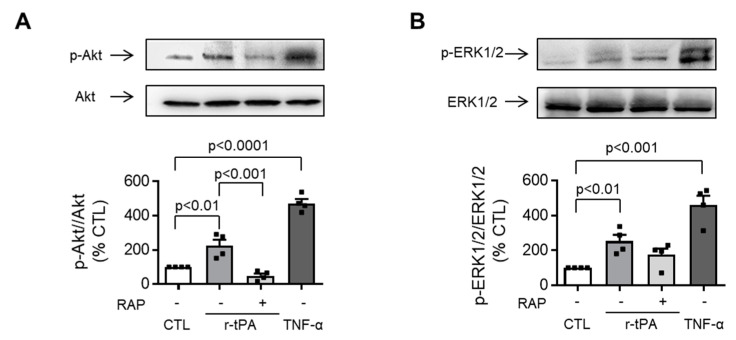
Pre-treatment with receptor-associated protein (RAP) inhibits PI3K/Akt phosphorylation induced by r-tPA while not affecting ERK1/2. Representative Western blot analysis and densitometric analysis of (**A**) Akt, and (**B**) ERK1/2 phosphorylation. Experiments were performed in polystyrene plates, cells were pre-treated for 1 h with 0.5 µM of RAP and stimulated with control medium (CTL) or 0.1 mg/mL r-tPA for 5 min, 200 U/mL of TNF-α was used as a positive control. Densitometric analysis was performed by dividing phospho-proteins to total amounts, afterwards data from the different group were normalized to their corresponding CTL and expressed as %. Mean ± 1 SEM, *n* = 4.

We previously demonstrated that incubation with r-tPA was able to induce the phosphorylation of Akt, and ERK1/2 kinases [7] and that the migration of neutrophils towards r-tPA depends on its binding to the LRP-1 receptor. Based on those observations, we pretreated the neutrophils with RAP (the LRP-1 receptor antagonist) and stimulated cells with r-tPA to identify which pathway was activated through the LRP-1 receptor. Both Akt and ERK1/2 were confirmed to be activated by r-tPA stimulation (Figure 4), yet RAP pretreatment selectively inhibited Akt phosphorylation while showing no significant effects on ERK1/2 (Figure 4A,B, respectively). TNF-α served as the positive control for Akt and ERK1/2 phosphorylation.

### 2.5. LRP-1 Mediates r-tPA-Dependent Neutrophil MMPs Degranulation under Adhesion Conditions

As a complement to our previous report of r-tPA-dependent neutrophil degranulation [7], here we investigated whether LRP-1 mediates the observed effects. To this end, we used neutrophil culture models mimicking both adhesion (polystyrene dishes) or suspension (Teflon^TM^ dishes) conditions in vitro [10]. We confirmed that r-tPA was able to induce the release of MMP-9, MMP-8 and MPO by degranulating neutrophils under both adhesion and suspension conditions (Figure S1A–C and Figure S1D–F, respectively). When pre-incubated with the LRP-1 inhibitor RAP, neutrophils under adhesion conditions showed a significantly reduced r-tPA-mediated release of MMP-9 and MMP-8 but not MPO, as compared with cells incubated with control medium (Figure S1A–C). On the opposite, in suspended cells RAP pre-treatment failed to reduce r-tPA-mediated release of MMP-9 and MPO, while a trend towards reduction was observed for MMP-8 although not statistically significant (Figure S1D–F). Phorbol myristate acetate (PMA) worked as a positive control for degranulation experiments.

## 3. Discussion

Here we showed that: (i) clinically relevant concentrations of r-tPA induce dose-dependent migration of human neutrophils by acting as chemoattractant and inducing chemokinesis via the activation of Akt intracellular pathway; (ii) r-tPA membrane receptor LRP-1 is synthetized and expressed by neutrophils in response to r-tPA and TNF-α; (iii) r-tPA-mediated F-actin polymerization and migration is LRP-1-dependent and happens through Akt but not ERK1/2; (iv) LRP-1 accounts for r-tPA-induced neutrophil degranulation when cells are under adhesion conditions.

Beside its major fibrinolytic role, tPA was shown to possess pleiotropic effects varying among different cells and conditions [11,12]. Its recombinant form remains the gold standard treatment for acute ischemic stroke leading to early revascularization and rescuing of part the ischemic tissue. Yet, compelling evidence from animal stroke models suggests r-tPA to have deleterious side effects favoring blood-brain barrier breakdown through increased endothelial and astrocyte expression of MMPs and increasing NMDAR-mediated neurotoxicity [13]. Furthermore, r-tPA was also shown to modulate inflammation by acting on different immune cells. t-PA promotes microglia activation in the brain via LRP-1 and annexin II [14,15,16], activated glial cells release most of this fibrinolytic enzyme with autocrine effects fueling a vicious loop with pro-inflammatory and neurotoxic effects [16,17]. Furthermore, tPA contributes to the adhesion and transmigration of monocytes and T-cells in vitro through NMDAR and LRP-1 mediated signaling [18,19]. Yet, whether tPA also leads to the pro-inflammatory activation of macrophages remains to be fully elucidated with different reports showing heterogeneous results [20,21]. Similarly, also reports on the effects of tPA and r-tPA on neutrophils not always show coherent results. In animal models of ischemia and ischemia/reperfusion injury, tPA promotes postischemic neutrophil recruitment via different mechanisms including proteolytic activation of plasmin and MMPs as well as increase microvascular leakage, yet tPA does not seem to induce neutrophil chemotaxis in murine cells on its own [22,23,24]. In the clinical setting, we previously showed that the circulating levels of neutrophil degranulation products (MMP-8, MMP-9, MPO and NE) peak in stroke patients receiving r-tPA during the first hour after drug administration [7]. Further, we demonstrated that, in vitro, r-tPA induces neutrophil degranulation in both adherent and suspended cells through Akt and ERK1/2 intracellular signalling [7]. Yet, the specific effects of r-tPA on human neutrophil functions other than degranulation remained to be investigated. Here, we complemented our previous report by showing a direct dose-dependent chemotactic effect of r-tPA on human neutrophils. In addition, by mean of kinase inhibitors, we demonstrate a role for PI3K/Akt pathway rather than ERK1/2 in this effect. Thus, tPA released by activated or dying resident immune cells in the stroke area likely plays a dual role in attracting circulating neutrophils in the ischemic parenchyma as well as inducing their degranulation thereby contributing to the deleterious effect of those cells in the early stroke evolution. Of interest, when percutaneous coronary intervention (PCI) is not an immediate option, current guidelines on ST-elevated myocardial infarction (STEMI) recommends fibrinolytic therapy with r-tPA [25]. Yet, a recent trial investigating the use of low-dose r-tPA as an adjunctive therapy to PCI was stopped because of futility [26], with a post-hoc analysis demonstrating that r-tPA could potentially increase microvascular obstruction in STEMI patients with ischemic time between 4 to 6 h [27]. As neutrophils play an important role in the pathophysiology of such a complication, we can speculate that r-tPA-mediated neutrophil degranulation and migration might have contributed to the neutral findings of the trial. This hypothesis requires confirmation by specific studies. 

LRP-1, also known as alpha-2-macroglobulin receptor or apolipoprotein E receptor, is a membrane protein belonging to the family of low-density lipoprotein receptors [9]. LRP-1 works as a scavenger and signaling receptor in several physiological and pathological processes through modulation of receptor-mediated endocytosis. t-PA binds to and activates LRP-1 alone or in complex with PAI-1 with different affinities. In accordance with a single previous report [28], we herein confirmed that human neutrophils express *LRP1* mRNA and expose the corresponding protein on their membrane. Furthermore, we demonstrated that both r-tPA and the neutrophil strong activator TNF-α can induce an upregulation of *LRP1* transcription resulting in increased levels of protein exposure on the cellular membrane. These results suggest a positive feedback of inflammation and r-tPA stimulation on neutrophils which can fuel itself and perpetuate its deleterious role in different conditions including ischemic stroke. In order to investigate whether LRP-1 was the mediator of the r-tPA-induced neutrophil migration we employed RAP that occupies LRP-1 binding sites and prevents r-tPA-mediated signaling activation [9]. In line with our hypothesis, pretreatment with RAP significantly inhibited F-actin polymerization and neutrophil migration in response to r-tPA, but not CXCL8. Of interest those results are in line with a previous report showing that blockade of LRP-1 prevents intravascular adherence and neutrophil recruitment within the ischemic tissue [29]. Furthermore, in the same work the authors found that exogenously administrated PAI-1 acts as an inflammatory mediator that guide circulating neutrophils to postischemic tissue through LRP-1 [29]. In consideration of our results and the previously showed high affinity of tPA and PAI-1 towards LRP-1 when in complex [9], we cannot exclude that those observations were at least partially mediated also by tPA. Furthermore, we complemented our previous report on the effect of r-tPA on neutrophil degranulation [7] by showing a role for LRP-1 in mediating this effect at least in cells under adhering conditions. Lastly, we confirmed the important role of PI3K/Akt in r-tPA-mediated neutrophil chemotaxis by showing that LRP-1 inhibition by RAP could specifically prevent Akt, but not ERK1/2, phosphorylation in response to r-tPA. Again, those results are in line with the available literature showing the involvement of PI3K/Akt pathway in the intracellular transduction of r-tPA signaling via LRP-1 in cells other than neutrophils [13,15].

Some limitations need to be acknowledged. Firstly, we could not investigate the transcription factors involved in r-tPA-mediated upregulation of *LRP1* transcription in neutrophils. Yet, p53 seems to be a likely candidate given the previous evidence showing its involvement in LRP-1/Akt transduction pathway as well as its regulatory role on such gene [30,31]. Furthermore, we cannot exclude the involvement of receptors other than LRP-1, in particular for r-tPA-mediated neutrophil degranulation. MMPs hold detrimental effects in the context of brain ischemia/reperfusion injury and have been long time indicated as possible therapeutic target [32]. As MMP-8 was shown to play beneficial roles during the healing process [33], further studies should assess the long-term effects of neutrophil inhibition in the context of ischemic stroke. Finally, r-tPA effects on other ischemia/reperfusion-related neutrophil functions, such as phagocytosis and neutrophil extracellular traps formation, were not addressed in this research work and are the goals of our future research in the field.

## 4. Materials and Methods 

### 4.1. Isolation of Human Primary Neutrophils

This protocol was approved by the local Ethics Committee of San Martino Hospital in Genoa (Italy) and conformed to the principles outlined in the Declaration of Helsinki. Neutrophils were isolated from human heparinized (Heparin 10 U/mL; Pfizer Pharma GmbH, Berlin, Germany) venous blood from healthy volunteers (age 24–48 years old) after written informed consent. Neutrophils were prepared by Dextran 70.000 (Plander, Fresenius Kabi Italia, Verona, Italy) sedimentation, followed by centrifugation (400× *g*, 30 min) on a Ficoll-Hypaque (Cedarlan Laboratories Ltd., Ontario, Canada) density gradient. Plasma and the mononuclear cell ring were discarded, and contaminant erythrocytes were removed by hypotonic lysis [34]. Neutrophils were then re-suspended in culture medium and used for different experiments as hereby described. Neutrophils were 97% pure on average, as determined by morphological analysis of May-Grünwald Giemsa (Merck, Darmstadt, Germany) stained cytopreparations [10].

### 4.2. Modified Boyden Chamber Migration Assay and Checkerboard Analysis

After isolation, neutrophils were diluted in the migration medium HBSS (EuroClone, Wetherby West, UK) containing 1 mg/mL bovine serum albumin (BSA, from Sigma-Aldrich, St. Louis, MO, USA) and tested for migration towards r-tPA. Chemotaxis assay was performed be mean of a 48-well microchemotaxis chamber (Neuro Probe Inc., Gaithersburg, MD, USA) using a 3 μm-pore size, with a 150 μm thick cellulose ester filter (Neuro Probe Inc.) [35]. The lower wells of the chemotaxis chambers were filled with either control medium alone or different concentration (up to 1 mg/mL) of r-tPA, 1nM human recombinant CXCL8 (BioSource International, Camarillo, CA, USA) was used as positive control. The upper wells were filled with 1 × 10^5^ neutrophils suspended in 50 μL. After 30 min-long incubation at 37 °C and 5% CO_2_, the filters were removed from the chambers, washed and stained with Harris hematoxilin (Baxter, Rome, Italy). After dehydration, the filters were cleared with xylene and mounted in Eukitt (Kindler, Freiburg, Germany). Each condition was performed in duplicate. The migration, as the distance (µm) travelled by the leading front of cells was measured at 1000× magnification. Data were expressed as migration towards control medium, r-tPA or CXCL8. In a set of experiments, neutrophils were pre-incubated (1 h) with intracellular kinase inhibitors [10 µM LY294002 (PI3K inhibitor; Sigma-Aldrich), 10 µM U0126 (selective inhibitor of MEK 1/2; Sigma-Aldrich)] or 0.5 µM of the low density lipoprotein receptor-related protein 1 (LRP-1) inhibitor, receptor-associated protein (RAP, Oxford Biomedical Research Inc, Oxford, UK). At the end of incubation time, cells were washed and tested for chemiotaxis towards 0.03 mg/mL r-tPA or 1 nM CXCL8 [36].

Checkerboard analysis was performed by adding different doses of r-tPA (0, 0.03, 0.1, 0.3 mg/mL) both in the upper (with the cells) and in the lower well. After incubation at 37 °C for 1 h, the filters [5 μm-pore size, polycarbonate filter (Neuro Probe Inc.)] were then removed from the chambers, washed and stained with Diff-Quick (Baxter) [37]. Each experimental condition was performed in duplicate by a blinded observer. The cells of five random oil-immersion fields were counted and the chemotaxis index was calculated from the number of cells migrated to the test samples divided by the number of cells migrated to the control (chemotaxis index).

### 4.3. Semi-Quantitative and Real Time (RT)-PCR

Total RNA was extracted by UPzolTM RNA Isolation Solution (Biotechrabbit TM GmbH, Hennigsdorf, Germany) from human neutrophils exposed to medium alone or 0.1 mg/mL r-tPA and 200 U/mL TNF-α for 2 h.

RNA concentration and integrity were evaluated on a NanoDrop ND-1000 Spectrophotometer (NanoDrop Technologies Inc., Wilmington, DE, USA). 500 ng RNA was used for cDNA synthesis with iScript cDNA synthesis kit (Biorad, Segrate, Italy). PCR amplification was carried out in a total volume of 10 μL, containing 1 μL cDNA solution, 5 μL PrecisionPLUS 2x qPCR MasterMix with Syber green (Primerdesign, Southampton, UK), 0.5 μL each primer (TIB MOL BIOL, Genoa, Italy), 3.5 μL of nuclease-free water. β-actin was quantified and used for the normalization of expression values of LRP1 gene. Assays were run in triplicate with Universal PCR Master Mix on MasterCycler realplex (Eppendorf) PCR system. PCR product was analyzed on agarose gel stained with ethidium bromide and visualized by ultraviolet illumination. Following primers have been employed: human *LRP1* For: ACCACCCCTCCCGCCAGCCCA, Rev: AGCCCGAGCCGTCGCCTTGC; human β-actin For: CATCCCCCAAAGTTCACAAT, Rev: AGTGGGGTGGCTTTTAGGA.

### 4.4. Immunocytochemistry

Neutrophil suspensions (1 × 10^6^/mL) were cytospun on culture glass slides positively charged. Then slides were fixed in cold methanol and staining was performed after quenching of endogenous peroxidase with 3% H_2_O_2_ in methanol. Slides were incubated with primary antibody overnight, followed by incubation with biotinylated antibody for 30 min. Each sample was analyzed for the detection of LRP-1 (Santa Cruz Biotechnology, Santa Cruz, CA, USA) and a labelled polymer HRP anti-mouse from Dako was used as secondary antibody. Staining was completed using the streptavidin-peroxidase method and DAB (3,3′-Diaminobenzidine) (Abcam, Cambridge, UK) [34]. Cytospins were counterstained with hematoxylin and mounted in Eukitt (Merck Group, Darmstadt, Germany), examined by light microscopy (Leica, Cambridge, UK), and evaluated by image analysis (Leica Q500 MC Image Analysis System, Leica). Isotype-matched IgG monoclonal antibodies (Santa Cruz Biotechnology) was tested as a negative control.

### 4.5. Flow Cytometry

After isolation, neutrophils were stained with phycoerythrin (PE)-conjugated anti-LRP-1 antibody (BD Pharmingen San Diego, CA, USA) or with isotype-matched, PE-conjugated IgG antibody (BD Pharmingen San Diego) as negative control. In selected experiments, neutrophils were treated with either medium alone (negative control), 200 U/mL TNF-α (positive control) or r-tPA (0, 0.03, 0.1, 0.3 mg/mL) for 20 min. After washing with PBS, cells were immediately stained with phycoerythrin (PE)-conjugated anti-LRP-1 antibody. All samples were analyzed on a FC500 (Beckman Coulter, Hialeah, FL, USA). CXP software was used for the acquisition and analysis. Autofluorescence levels of sample were measured and subtracted from each analysis. Results were expressed as median fluorescence intensity (MFI) -LRP-1 (70% CD91/LRP-1positive cells) [10].

### 4.6. Determination of the Total Cellular F-Actin

Freshly isolated neutrophils were pretreated for 1 h with or without 0.5 µM RAP. After this incubation time, cells were stimulated with either medium alone, 0.03 mg/mL r-tPA or 1 nM CXCL8 for additional 30 min. Then, neutrophils were prepared by cytospin centrifuge onto slides. After fixing and treatment with Triton X 100, cells were stained with Alexa-Fluor 488-conjugated phalloidin (Thermofisher Scientific, Waltham, MA, USA). F-actin was determined by fluorescence microscopy (Nikon Optiphot-2; Nikon, Melville, NY, USA), as previously described [38]. Image capturing was performed with a Hamamatsu color-chilled 3 CCD camera. All images were captured using identical camera settings (time of exposure, brightness, contrast and sharpness) and an appropriated white balance was set according to the fluorescence filter. Pictures were acquired and analyzed by Image-Pro Plus 4.0 (Media Cybernetics Inc., Rockville, MD, USA). The mean fluorescence density was determined from a linear measurement of individual cell fluorescence in randomly chosen fields of each slide. Results are shown as mean ± SEM of the fluorescence densities (expressed as the relative intensity of each field for each individual slide).

### 4.7. Western Blot Analysis 

After isolation, neutrophils (1 × 10^7^ cells/mL) were pre-incubated with RAP 0.5 µM for 1 h at 37 °C in a humidified atmosphere with 5% CO_2_ in polystyrene plates to assure adherence conditions. Then, cells were treated with either medium alone (negative control) or stimulated with 0.1 mg/mL r-tPA or 200 U/mL TNF-α (positive control, R&D Systems, Bio-Techne, Minneapolis, MN, USA) for 7 min. Cell activity was stopped on ice and the cells were centrifuged at 4 °C. After removing supernatants, the pellets were lysed in 400 μL of Nonidet P40 buffer (20 mM Tris–HCl pH 7.5, 0.15 M NaCl, 10 mM NaF, 1% Nonidet P40, 10 μg/mL glycerol, 1 mM phenylmethanesulphonyl-fluoride, 10 μg/mL leupeptin, 10 μg/mL aprotinin, 0.5 mM Na3VO4). Equal amounts of protein (20 μg for for each sample) were boiled in loading buffer (62.5 mM Tris–HCl pH 6.8, 0.75% SDS, 8.75% glycerol and 0.025% bromophenol blue) and resolved by 10% SDS–polyacrylamide electrophoresis. Then, proteins were transferred on nitrocellulose membrane at 4 °C for 45 min. After blocking 1 h in 5% non-fat dry milk and washing with Tris-buffered saline/Tween 20 (10 mM Tris-base pH 7.4, 154 mM NaCl and 0.05% Tween 20), membranes were incubated with appropriate dilution of anti-phospho-Akt (Santa Cruz Biotechnology), anti-phospho-ERK1/2 (R&D Systems) primary antibodies, as well as corresponding secondary antibodies. Blots were developed using the ECL system (Immobilion Western, Merck Group). Membranes were then stripped, re-blocked and re-probed to detect total intracellular kinases by using anti-Akt (Santa Cruz Biotechnology) or anti-ERK 1/2 (R&D Systems) primary antibodies. Immunoblots were scanned and quantifications was carried out by Image Quant software version 3.3 (Molecular Dynamics, Sunnyvale, CA, USA). Values of phospho-Akt, phospho-ERK 1/2, and phospho-p38 MAPK were normalized to corresponding total amounts of Akt, ERK 1/2 and p38 MAPK and expressed as percentages of negative control (defined as 100%).

### 4.8. Neutrophil Degranulation Assay

After isolation, 5 × 10^5^ neutrophils were incubated with control medium (serum-free RPMI 1640 medium containing 25 mmol/L HEPES for degranulation assay), 10 ng/mL PMA (positive control; Sigma-Aldrich) or 0.1 mg/mL r-tPA (Boehringer, Ingelheim, Germany) for 30 min at 37 °C in a humidified atmosphere with 5% CO_2_. Experiments were run in in Teflon^TM^ dishes to maintain cell suspension mimicking circulating conditions or under adherence conditions in polystyrene plates [10]. In selected experiments, neutrophils were pre-incubated for 1 h with control medium either without or with RAP (0.5 µM). Finally, supernatants were collected for the assessment of neutrophil degranulation products. 

### 4.9. Biomarker Measurement in Neutrophil Supernatants

Levels of MMP-8, MMP-9, and MPO in neutrophil supernatants were measured by colorimetric enzyme-linked immunosorbent assay (ELISA), following the manufacturer’s instructions. The lower limits of detection were 0.156 ng/mL for MMP-8, 0.312 ng/mL for MMP-9 and 0.156 ng/mL for MPO. Mean intra- and interassay coefficients of variation were <8% for all markers. 

### 4.10. Statistical Analysis 

Values are expressed as means ± SEM. One-way ANOVA with Tukey correction for multiple testing was used for multiple group comparison, while the unpaired Student t-test was used for comparison between two groups. Values with *p* < 0.05 (two-tailed) were considered significant. All analyses were performed with the GraphPad Prism statistical software, version 7 (GraphPad Software Inc., San Diego, CA, USA).

## 5. Conclusions

In conclusion, this study shows that the thrombolytic drug r-tPA induces neutrophil chemotaxis through LRP-1/Akt pathway. Similarly, also neutrophil degranulation in response to r-tPA is likely mediated by LRP-1 under adhesion conditions. Given the recognized deleterious role of neutrophil activation in the early post-ischemic phase of stroke, blunting r-tPA-mediated neutrophil activation might be a promising strategy as an adjuvant therapy to thrombolysis in acute ischemic stroke patients.

## Supplementary Materials

Supplementary materials can be found at https://www.mdpi.com/1422-0067/21/19/7014/s1.

## Abbreviations

CXCL8	C-X-C Motif Chemokine Ligand 8
ELISA	enzyme-linked immunosorbent assay
I/R	Ischemia/reperfusion injury
LDL	Low-density lipoprotein
LRP-1	Low density lipoprotein receptor-related protein 1
MFI	Mean fluorescence intensity
MMP	Matrix metalloproteinases
MPO	Myeloperoxidase
NE	Neutrophil elastase
PMA	Phorbol myristate acetate
RAP	Receptor-associated protein
r-tPA	Recombinant tissue-plasminogen activator
SEM	Standard error of the mean
TNF	Tumor necrosis factor
