# Supplementary material for: Recombinant Tissue Plasminogen Activator (r-tPA) Induces In-Vitro Human Neutrophil Migration via Low Density Lipoprotein Receptor-Related Protein 1 (LRP-1)
Source: Int J Mol Sci. 2020 Sep 23;21(19):7014. doi: 10.3390/ijms21197014 (PMC7582901; doi:10.3390/ijms21197014)
Supplement: Supplementary file 1 [file ijms-21-07014-s001.pdf]

Supplementary material

Recombinant tissue plasminogen activator (r-TPA) induces *in-vitro* human neutrophil migration via low density lipoprotein receptor-related protein 1 (LRP-1)

Luca Liberale[1,2]*, Maria Bertolotto[2]*, Silvia Minetti[2], Paola Contini[3], Daniela Verzola[4], Pietro Ameri[5,6], Giorgio Ghigliotti[5,6], Aldo Pende[5,7], Giovanni G. Camici[1], Federico Carbone[2,5], Fabrizio Montecucco[2,5]

[1] Center for Molecular Cardiology, University of Zürich, Wagistrasse 12, 8952 Schlieren, Switzerland. [2] First Clinic of Internal Medicine, Department of Internal Medicine, University of Genoa, 6 Viale Benedetto XV, 16132 Genoa, Italy. [3] Clinical Immunology, Department of Internal Medicine, University of Genoa, 6 viale Benedetto XV, 16132 Genoa, Italy. [4] Division of Nephrology, Dialysis and Transplantation, Department of Internal Medicine, University of Genoa, 6 Viale Benedetto XV, 16132 Genoa, Italy. [5] IRCCS Ospedale Policlinico San Martino Genoa – Italian Cardiovascular Network, 10 Largo Benzi, 16132, Genoa, Italy. [6] Laboratory of Cardiovascular Biology, IRCCS Ospedale Policlinico San Martino & Department of Internal Medicine, University of Genoa, Genoa, Italy. [7] Clinic of Emergency Medicine, Department of Emergency Medicine, University of Genoa, Genoa, Italy.

* These authors equally contributed as first author.

Running title: r-TPA favours neutrophil migration

Corresponding Author: Fabrizio Montecucco, MD, PhD, First Clinic of Internal Medicine, Department of Internal Medicine, University of Genoa, 6 viale Benedetto XV, 13132 Genova. Tel: +39 010 353 8694; Fax: +39 010 353 8686; E-mail: fabrizio.montecucco@unige.it

Supplementary figure1

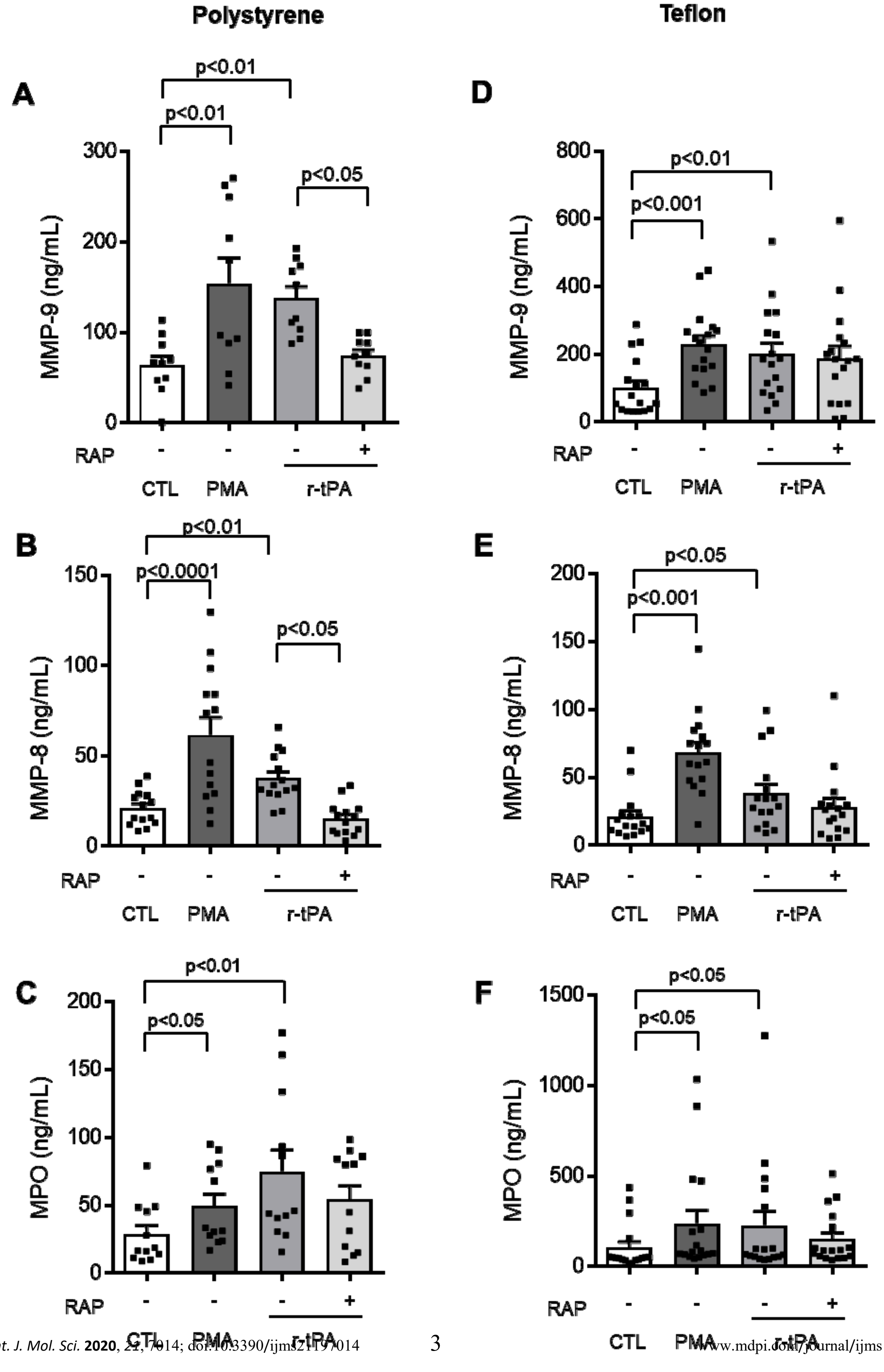

Suppl. Figure 1: Pre-treatment with receptor-associated protein (RAP) reduces recombinant tissue plasminogen activator (r-tpA)-induced human neutrophil degranulation under adhesion conditions. Cells under adherent (polysterene dishes, **A-C**) or suspension (Teflon™ dishes, **D-E**) were pre-treated for 1 hour with control medium (CTL) or 0.5 µM RAP and then stimulated with 0.1 mg/mL r-tPA or 10 ng/mL phorbol myristate acetate (PMA) as positive control for 30 minutes. Degranulation products metalloproteinases (MMP)-9 (**A, D**), MMP-8 **(B, E)** and myeloperoxidase MPO **(C, F)** were quantified by ELISA in the cell surnatants. Data are expressed as mean ± 1SD, n=11-17.
